# The First 3D Model of the Full-Length KIT Cytoplasmic Domain Reveals a New Look for an Old Receptor

**DOI:** 10.1038/s41598-020-62460-7

**Published:** 2020-03-25

**Authors:** François Inizan, Myriam Hanna, Maxim Stolyarchuk, Isaure Chauvot de Beauchêne, Luba Tchertanov

**Affiliations:** 1 Université Paris-Saclay, ENS Paris-Saclay, CNRS, Centre Borelli, 4 avenue des Sciences, F-91190 Gif-sur-Yvette, France; 2 Université de Lorraine, LORIA (CNRS, INRIA), F-54000 Nancy, France

**Keywords:** Computational models, Protein structure predictions

## Abstract

Receptor tyrosine kinases (RTKs) are key regulators of normal cellular processes and have a critical role in the development and progression of many diseases. RTK ligand-induced stimulation leads to activation of the cytoplasmic kinase domain that controls the intracellular signalling. Although the kinase domain of RTKs has been extensively studied using X-ray analysis, the kinase insert domain (KID) and the C-terminal are partially or fully missing in all reported structures. We communicate the first structural model of the full-length RTK KIT cytoplasmic domain, a crucial target for cancer therapy. This model was achieved by integration of *ab initio* KID and C-terminal probe models into an X-ray structure, and by their further exploration through molecular dynamics (MD) simulation. An extended (2-µs) MD simulation of the proper model provided insight into the structure and conformational dynamics of the full-length cytoplasmic domain of KIT, which can be exploited in the description of the KIT transduction processes.

## Introduction

Receptor Tyrosine Kinases (RTKs) are cell-surface transmembrane proteins that control cell-signalling pathways^1^_._ They act as sensors for extracellular ligands, the binding of which trigger receptor dimerization, activation of the cytoplasmic domain and intermolecular autophosphorylation of specific tyrosine residues. These intra-receptor processes lead to the recruitment, phosphorylation and activation of multiple downstream signaling proteins, which eventually govern the cell physiology^2^. RTK mutations and aberrant downstream signaling have been linked to many diseases^3^, while pharmacological modulation of RTKs activation has been successfully used in the treatment of a wide range of cancers^4^.

**Figure 1:**
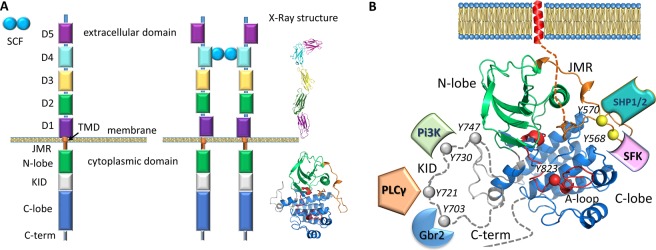
Structure of KIT, a member of the RTK class III. (**A**) Structural composition of KIT: an extracellular domain (ECD) with five Ig-like regions (D1–D5), a transmembrane domain (TMD) and a cytoplasmic domain (CD) comprising a juxtamembrane region (JMR), an ATP-binding region (N-lobe), the phosphotransferase domain (C-lobe) spliced by a kinase insert domain (KID) and a C-terminal. Stem Cell Factor (SCF) extracellular binding induces dimerization and activation of KIT. Column ‘X-Ray structure’ represent the crystallographic structures of ECD (PDB: 2CE8^52^) and CD (PDB: 1T45^20^) of KIT. (**B**) Structure of the KIT CD (PDB:1T45) with missing KID, JMR N-term and C-terminal fragment as pointed lines. The tyrosine residues (Y) and the proteins that specifically recognize the KIT phosphotyrosine sites are schematized.

A first vision of RTKs activation molecular mechanisms was developed from structural studies, principally performed by X-ray analysis^5,6^. The structure of type III RTKs consists of an extracellular ligand-binding domain formed by five Ig-like fragments connected to a cytoplasmic domain (CD) by a transmembrane helix (Fig. 1A). The cytoplasmic region contains a tyrosine kinase (TK) domain that is composed of proximal (N-) and distal (C-) lobes linked by a kinase insert domain (KID) of varied length among the RTKs^7^. The catalytic mechanism involves fragments from the TK domain that regulate RTK activity (the activation (A-) loop, the juxtamembrane region (JMR) and the Cα-helix), while the post-transduction processes, phosphorylation and the intracellular protein binding, are associated with the JMR, the KID and the C-terminal, which are the regions possessing multiple phosphorylation sites^2^.

A study of the transduction processes in RTKs using experimental techniques is difficult and/or highly restricted. A theoretical description at structural level is also limited or impossible because no experimental data describing a full-length RTK falls short of revealing the atomic structural details of the long-length KID^8,9^. A considerable number of structures characterizing RTKs have been published, but they report only partial fragments (Fig. 1). The CD of platelet-derived growth factor receptor (PDGFR) and vascular endothelial growth factor receptor (VEGFR) families (types II and IV RTKs) was extensively studied by X-ray analysis that demonstrated the structures of the native and mutated forms in the active and inactive states, representing the free-ligand or ligand-bound species. However, the KID, N-terminal of JMR and C-terminal (C-term) residues are partially or fully missing in all these structures (Fig. 1). The KID, which interrupts the kinase domain in the majority of RTKs (38 out of 58), was systematically deleted or replaced by a short pseudo-KID *prior* to crystallization of RTKs from the PDGFR and VEGFR families, an option adapted from the *in vitro* studies showing that KID does not influence the kinase activity^10^ and its deletion does not affect the overall structure of CD^11^ nor the binding of inhibitors in its active site^12^.

The KID sequence is strongly varied in length and amino acids (aas) composition. Receptors from the PDGFR and the VEGFR families are characterized by a long KID (62–97 aas), while other receptors contain a shorter (10–23 aas) or tiny KID (4–9 aas)^7^. There is no detectable sequence homology between the KIDs of receptors from different families: sequence homology is only noticeable within the same type of family. This poor sequence homology of KID (13–31% for type III RTKs, a group made of KIT, CSF-1R, FLT3, PDGFR-α and PDGFR-β) with respect to the TK domain (61–76% for type III RTKs) delivers a benchmark for delimiting these two domains. In contrast to the short KIDs that are frequently free of Ser/Thr/Tyr residues that enable them to be phosphorylated, the long KIDs are highly populated by the phosphorylation sites, and therefore are indispensable for downstream signaling by the activated kinase. In KIT, a unique RTK that has Tyr and Ser residues simultaneously as functional sites, the KID (76 aas) contains five functional phosphorylation sites, three tyrosine (Y703, Y721, Y730), and two serine (S741 and S746), which provided the alternative binding sites for signaling or adaptor proteins [^7^and references herein]. Phosphorylation of Y703 furnishes the binding site for the SH2 domain of Grb2, an adaptor protein initiating the Ras/MAP kinase signaling pathway (Fig. 1B). Y721 and Y730 are the recognition sites of PI3K and phospholipase C (PLCδ). The function of Y747 is not yet described. Phosphorylated S741 and S746 bind PKC (protein kinase C) and contribute to re-control of PKC activity under the receptor stimulation. Similarly, the C-terminal is systematically absent in crystallographic structures and it contains the functional phosphotyrosine Y936, which forms the primary association site for adaptor proteins, Grb and APS^13^.

Here we report the first 3D model of the full-length KIT cytoplasmic domain in the inactive state and its study using an extended (2-µs) molecular dynamics (MD) simulation. This *in silico* conceived model, with maintained tyrosine residues in KID and C-terminal, delivers a structural platform for the exploration of phosphorylation effects, opening up routes for the study of the KIT post-transduction process, in particular the interaction with signalling or adaptor proteins.

## Results

### Modelling the full-length KIT cytoplasmic domain

Progress in computational algorithms and technique has enabled in depth study of protein molecular structure and dynamics using limited experimental data^14–17^. The model is built based on a known 3D homologous protein structure is at present the widely used approach. Since KID and the C-terminal are systematically absent in all RTK crystallographic structures reported in the PDB^6^, the template-based prediction of their structure is impossible. We suggest that in KIT, the large KID and C-terminal may have an intrinsic folding and different positions with respect to the kinase domain, which would explain their absence in the crystal structures. To test this hypothesis, we have first predicted the secondary structure of KID and C-terminal and estimated the degree of disorder.

**Figure 2:**
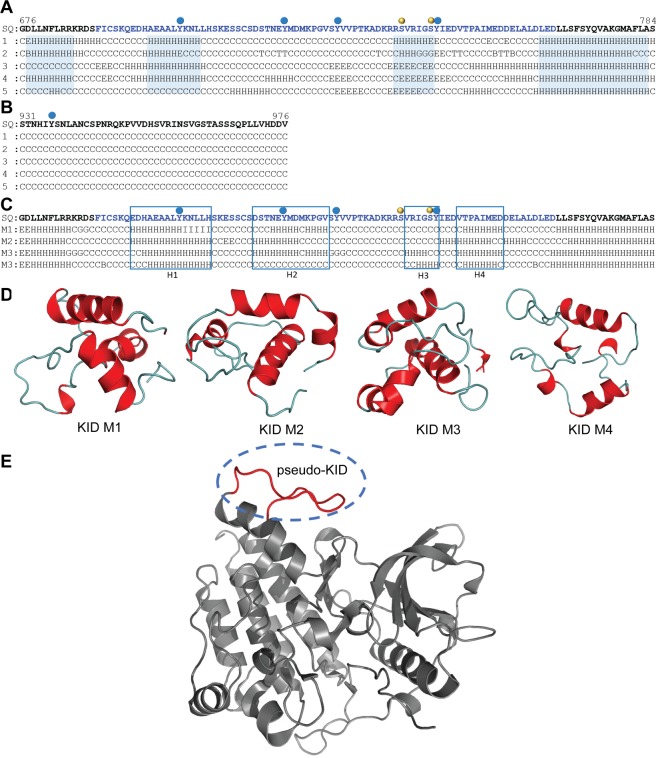
Modelling of the full-length KIT cytoplasmic domain. Prediction of the secondary structure content of KID (**A**) and C-term (**B**). The KID sequence and its folded regions are displayed in blue. Symbols ‘H’, ‘G’, ‘E’, ‘B’ and ‘C’ denote of α-helix, 3_10_-helix, β-strand, β-bridge and coil respectively. (**C**) Alignment of secondary structure in KID models **M1**‒**M4**. Positions of helices H1-H4 are framed. (**A–C**) Phosphorylation sites are indicated by red (tyrosine) and yellow (serine) balls. The KID *de novo* models (**D**) were integrated into structure 1T45 (**E**, in grey) by replacing the *pseudo*-KID (in red).

As the KID sequence length is not strictly delimited^18^, for the secondary structure prediction we used a *sensu largo*s sequence G676–S784 that includes the KID and 10 residues from each lobe (N- and C-lobes) of the KD. Using five bioinformatics methods based on distinct algorithms (see Methods), we found that some KID fragments are predisposed to form a regular structure (Fig. 2A). Despite the high level of disorder in KID (40–100%), we suggest a regular folding that involves at least three sequence fragments, while the C-terminal is apparently arranged as a random coil (Fig. 2B).

**Figure 3:**
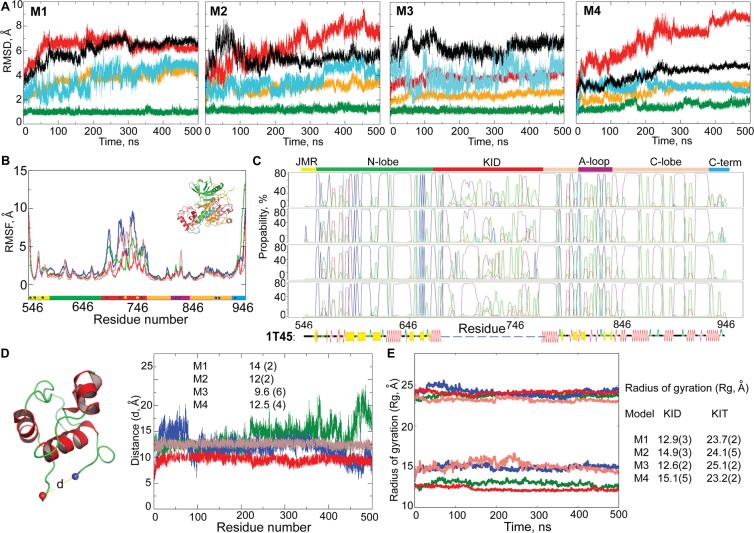
Molecular dynamics simulations of candidate models **M1–M4**. (**A**) RMSDs from the initial coordinates computed for all Cα-atoms (black), then only Cα-atoms from N-lobe (green), C-lobe (orange), KID (red) and C-term (blue light) for the **M1**–**M4** MD simulations. (**B**) RMSFs computed on the Cα atoms for MD conformations of each model − **M1** (green), **M2** (blue), **M3** (red) and **M4** (brown). A colour-coded bar delimits the KIT structural domains. (**C**) Secondary structure (SS) assignments for KIT models during MD simulations. For each residue, the proportion of SS type is given as a percentage of the total simulation time and shown with coloured lines: α-helix in violet, 3_10_-helix in red, parallel and antiparallel β-sheet in blue and cyan, turn in green. At the bottom: SS of 1T45 is interpreted by DSSP. (**D**) Distance (d) between residues F689 and D768 and (**E**) radius of gyration (Rg) over the MD simulation of models **M1** (green), **M2** (blue), **M3** (red) and **M4** (salmon) and their mean and standard deviation values.

A representative structure **M** from each cluster was selected, visually analyzed (PyMol) and further studied using MD simulations. In all representative models, the KID consists of either three or four helices (H1, H2, H3 and H4), which were expected from the secondary structure prediction (Fig. 2C). The principal differences between these models consist of (i) the number of helices in the KID, (ii) the length of the helices and their relative orientation in space, and (iii) the C-term position (Fig. 3D).

To build 3D models of the full-length KIT cytoplasmic domain, we used a hybrid protocol that combines *de novo* techniques (Rosetta and MODELLER) and crystallographic data (PDB: 1T45) (see Methods). From a large set of generated *ex situ* 3D KID models (2,000 models), 70 items with a distance between residues F689 and D765 close to the experimentally observed value (of 9.9 ± 1.0 Å), were inserted into structure 1T45 to replace the *pseudo*-KID (Fig. 2D,E). After completing the KIT with the *ab initio* structure of C-term, the models without structural aberrations (*i.e*., intramolecular ‘nodes’), having a good stereochemical quality and the highest DOPE scores (*i.e*., lowest energy) were grouped according to their secondary structure similarity.

### Identification of the most realistic model using MD simulation

Four candidate models (**M1**, **M2**, **M3** and **M4**) of the full-length KIT cytoplasmic domain were explored by all-atoms MD simulations. Since the success of MD simulations over a ten to hundreds of nanoseconds time scale for the refinement of homology or *ab initio* models of small to medium-size proteins was demonstrated^14,15,17,19^, we expected that the 500-ns MD simulations may be pertinent when scanning a conformational stability of the alternative KIT models. We suggest that such simulation time would allow the equilibrated state of the protein to be reached for a biologically relevant model. We then considered a comparison of the structure/dynamics–related metrics of candidates leading to a consensus result as the most appropriate strategy to establish the ‘most realistic model’.

First, each model was studied by two 100-ns MD simulations. The RMSD curves of N- and C-lobes in two trajectories of each model, **M1**, **M3** and **M4**, show very similar character, almost stable in **M3** and **M4** and showing a tendency to increase in **M1** (Fig. S1). Consequently, the simulations of each model were extended to 500 ns for only the one trajectory showing the smallest RMSD values. One trajectory of model **M2** (**b**, Fig. S1) shows a fast increasing of the C-lobe RMSDs in the range of 80–100 ns and very high RMSFs values in all domains. It was stopped and the other one was extended to 500 ns. The 500-ns MD trajectories of models **M1**–**M4** were used for selection of a candidate model.

The root mean square deviations (RMSDs) show significant variations (up to 8 Å) until ~300–350 ns, and further, reach a plateau for each simulated model (Fig. 3A). The *per domain* RMSDs revealed that the N-lobe was perfectly stable for each model. The C-lobe shows a small RMSD in only **M3**, while in **M1**, **M2** and **M4** it is either variable during the simulation time (**M1** and **M2)** or stabilised only in the second half of simulation (**M4**).

The RMSD profiles of KID and their values vary largely between the models and during the simulation of each model, reflecting a large variability of this domain within the simulation time, for **M2** and **M4**, and stability of **M1** and **M3** after 300 and 150 ns respectively. Comparing **M1** and **M3**, the RMSDs for C-lobe and KID are considerably lower in **M3**. The RMSD of C-terminal varies significantly in each model, except in **M4**. Finally, the structure of the kinase domain is better preserved throughout the MD simulation in **M3**, with the RMSD never exceeding 2.5 Å. The atomic root mean square fluctuation (RMSF) curves have similar profiles for all simulated models, but show lower values of **M3** (Fig. 3B).

Structure 1T45, where the distance *d* between F689 and D768 is 9.9 Å^20^, is a corner stone for the modelling of KIT. This ‘end–to–end’ distance is highly conserved over all published structures of KIT (Table 1). Apparently, its value is independent of KIT state (active or inactive), form (native or mutant) or protein binding (bound to ligand or not). This distance among type III RTKs is either similar (KIT, PDGFRα or FLT3) or different (14.2 Å in CRF1-R). We suggest that this distance is a specific metric for a given RTK and may be used as criteria for its structure correctness. The value of distance *d* over MD conformations varies little in **M3** and **M4**, and is considerably altered in **M1** and **M2** (Fig. 3D). Only the **M3** shows a distance *d* close to the experimental value.

**Table 1:** Receptors tyrosine kinase (RTKs) from III family characterized by X-ray analyses^6^.

RTK	PDB code	Resolution	Distance	State/form/ligand
KIT	1T45	1.9	9.9	Inactive autoinhibited
	1PKG	2.9	10.2	Active
	3G0E	1.6	10.1	Bound to sunitinib
	3G0F	2.6	10.6	Mutant bound to sunitinib
	1T46	1.6	10.0	Inactive non autoinhibited
CSF1-R	4R7I	2.75	14.2	Bound to imatinib
	4HW7	2.9	14.1	Bound to PLX647-OME
	4R7H	2.8	14.3	Bound to PLX3397
PDGFRα	5GRN	1.77	10.1	Bound to WQ-C-159
	5K5X	2.17	10.0	Canonical
FLT3	1RJB	2.1	10.2	Canonical
	5X02	2.4	10.4	Bound to FF-10101

Each structure referenced with the PDB code, structure resolution (Å), distance (Å) between F689 and D768 residues in KIT or between corresponding residues in CSFR-1, PDGFRα and FLT3, state/form of RTK.

Furthermore, we estimated the compaction of candidate models from the radius of gyration (Rg). During the MD simulation, the KID of **M3** shows the smallest fluctuations of Rg (mv of 12.6 (2) Å), indicating a compact and potentially stable structure (Fig. 3E).

To quantify the extent to which the generated KIT models represent a stable local structure for the studied time-scale, we assigned the secondary structures and compared them with those observed in the template structure 1T45. In general, the secondary structure of the N- and C-lobes is well maintained over the simulation of all models with respect to the template 1T45 (Fig. 3C). The largest disparity is observed in the C-lobe region adjacent to KID, evidenced as a reduction in length of the well-defined α-helix of C-lobe adjacent to D768 in **M1**, **M2** and **M4**. This partial unfolding apparently contributes to the higher RMSDs of C-lobe and to the enlarged distance *d* between F689 and D768. The secondary structure of the kinase domain (N- and C-lobes) is well preserved in **M3**.

Analysis of MD simulations of four candidate models built to represent the 3D structure of the full-length KIT cytoplasmic domain shows that the independent statistical techniques characterizing different structural metrics deliver coherent results. Namely, the RMSDs and RMSFs, secondary structure composition, radius of gyration and distance between F689 and D768 indicate that **M3** is the most probable model among the studied candidate models.

### Structure and dynamics of the full-length KIT cytoplasmic domain

To investigate the correctness of the **M3** model, we tested its MD simulation reproducibility. A second trajectory of 500-ns was generated for the same structural model under identical conditions using random starting velocities. Furthermore, to probe the conformational variability of the full-length KIT cytoplasmic domain in the inactive auto-inhibited state, one MD simulation of the model **M3** was extended to 2 µs.

### General MD simulation characterization

The RMSDs and RMSFs computed on two 500-ns MD replicas respective to the same initial conformation display comparable profiles, demonstrating their good reproducibility (Fig. S2). The distance *d* between F689 and D768, which is used as the control metric, is perfectly conserved in the two replica and gives similar values (mv of 9.6 (6) and 10.7 (4) Å).

**Figure 4:**
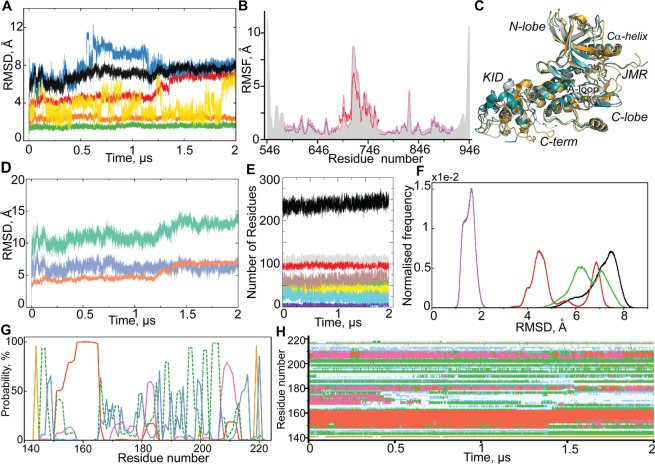
2-µs MD simulation of the M3 model of full-length cytoplasmic domain of KIT. (A) RMSDs from the initial coordinates computed for all Cα atoms (black), then only Cα atoms from N-lobe (green), C-lobe (orange), KID (red), JMR (yellow) and C-term (blue) after fitting on initial conformation. (**B**) RMSFs computed for all Cα atoms (grey contour), then only for Cα atoms from the partial protein composed of KD and KID (rose); the KD (violet) and the KID (red). (**C**) Superimposition of KIT conformations taken at t = 250 ns (teal), at 750 ns (grey) and at 1500 ns (orange). (**D**) RMSDs are individually calculated for KID for the data after the least-square fitting of the MD conformations on the initial conformations of KIT (‘all displacements’, in aquamarine) and KID (‘internal components’, in salmon) and the difference between them (in blue). (**E**) Secondary structure in KIT. The position of the curves reflects the amount of secondary structure elements in the models, from a tiny occurrence [π-helix (violet) and β-bridge (blue)] to a prevalent [α-helix (grey), coil (red) and turn (brown)], and sum of folded structures in black. (**F**) Conformational clusters grouped by RMSD (C*α*-atoms) with respect to the *first conformation*: the CD, composed of KD, KID, JMR and C-term (black); the two-domain region composed of KD and KID (green); the KD (red) and the KID (violet). Each histogram is normalized; 1000 bins are used. (**G**–**H**) Secondary structure assignment in KID (**G**) and its variations (**H**) over MD simulation: α-helix (red), 3_10_-helix (magenta), β-bridge (green), turn (yellow) and bend (blue).

Further analysis focuses principally on the extended (2-µs) MD trajectory. The *per domain* RMSD analysis demonstrates a high stability of both lobes of the kinase domain over the trajectory (Fig. 4A).

The RMSDs of JMR and C-terminal display a great variability over time. Remarkably, the RMSDs profile of the C-terminal shows two sudden changes, at 555-ns and at 1152-ns, suggesting a large change in its conformation or its position with respect to the KD. To check the suggested conformational transitions, we compared the MD conformations picked before and after each sudden RMSD change. In KIT conformations picked at t = 250, 750 and 1500 ns, the C-term is localized in close proximity to the KID, to the C-lobe and between these two positions, respectively (Fig. 4C). The strong variations in the JMR and C-terminal positions is reflected in their large root mean square fluctuations (RMSFs) (Fig. 4B). Surprisingly, the RMSD curve of KID shows good stability (mv of 4.5 Å) within the range of 5–1190 ns, then it incrementally increases in the range of 1190–1365 ns and then is again stabilize until the end of simulation (mv of 7 Å) (Fig. 4A).

### Conformational diversity of KIT and KID

In order to better describe these effects, the RMSDs were calculated for each domain, KID and KD, individually, and for a two-domain region (KID and KD) for the data after the least-square fitting of the MD conformations to the region of interest. Also, the RMSDs were computed independently of the size of the analyzed domain. This RMSD analysis showed that (i) the slope is observed for all KID RMSD curves obtained with different fitting procedures, (ii) the displacement of KID with respect to KD contributes to the large amplitude of RMSDs, however the inherent KID dynamics is the main factor contributing to the observed transition (Figs. 4D, S3). Similarly, the RMSFs computed for the two-domain KIT are only 25% greater than the values computed on the single KID (Fig. 4B).

Clustering of the conformations according to their RMSDs shows that the JMR, C-term and KID are the domains that contribute most to the conformational diversity of KIT (Fig. 4F). If JMR and C-term are excluded from consideration, the KIT conformations display a bimodal distribution that is mainly influenced by the KID.

Secondary structure analysis and a visual inspection of the MD trajectory from 1150 to 1450 ns (the transition range) shows that the transition between different KID configurations consists of the folding/unfolding of KID helices and the large movement of the KID fragments within the domain (Fig. 4G–H; Movie S1).

Note that the stereo chemical quality of the most flexible fragment of KID (S712–G727) shows that more than 96.5% of the residues (non-glycine/non-proline) were in allowed regions of the Ramachandran plot (Fig. S4).

### Intrinsic motion in KIT and its interdependence

**Figure 5:**
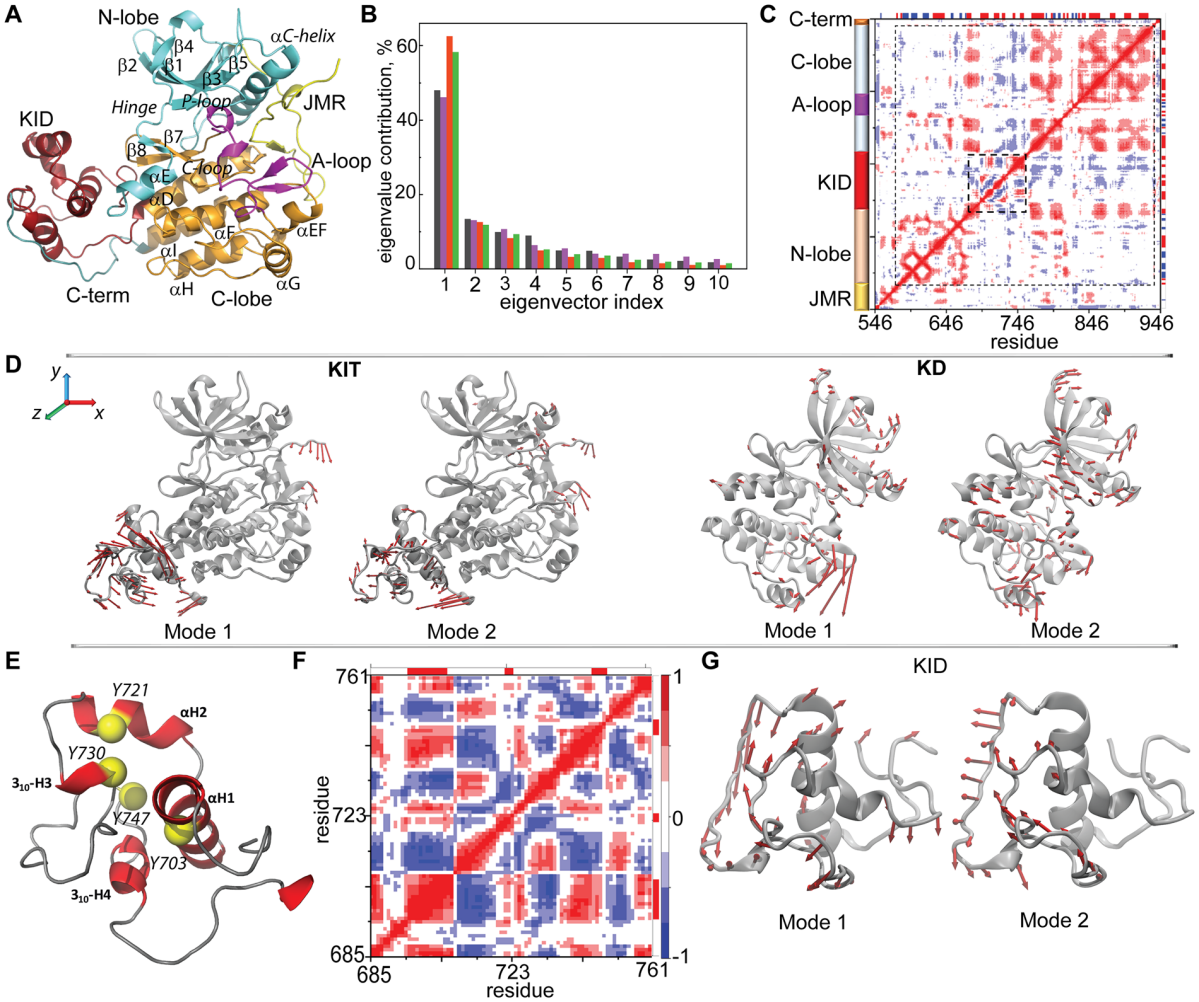
Intrinsic motion in KIT and its interdependence. (**A**) Cartoon of KIT with the labelled structural fragments (N-lobe, C-lobe, A-loop, JMR, KID, and C-term in turquoise, orange, violet, yellow, dark red and blue respectively). (**B**) The PCA modes calculated for each structural domain after least-square fitting of the MD conformations to the *average conformation* of the respective domain as a reference. The bar plot gives the eigenvalue spectra in descending order for the first 10 KIT modes (black), KD (violet), KID (red) and of the two-domain region (KD and KID, green). (**C**) Inter-residue cross-correlations map computed for all Cα-atom pairs of KIT. Dashed lines delimit KD and KID. Secondary structure projected onto the KIT sequences (α-helix/β-strand in red/blue). (**D**) Atomic components in PCA modes 1–2 are drawn as red arrows projected on the cartoon of KIT (left) and of KD (right). A *cutoff* distance of 1.5 Å (KIT) and 1.0 Å (KD) was used. (**E**) KID with the tyrosine residues shown as yellow balls. (**F)** Inter-residue cross-correlation map computed for the Cα-atom pairs of KID. (**G**) Atomic components in the PCA modes 1–2 are drawn as red arrows projected onto the KID. A *cutoff* distance of 1.5 Å was used. Correlated (positive) and anti-correlated (negative) motions between Cα-atom pairs are shown as a red-blue gradient.

To characterize this transition, we first explored the collective motion of KIT by the principal component analysis (PCA) after the fitting procedures based on a per domain (local) perspective. Ten PCA modes were sufficient to describe ~90% of the total backbone fluctuations of the KIT’s full-length CD and of its different structural domains (Fig. 5A,B).

The first two PCA modes of KIT reveal the essential mobility of JMR, KID and C-terminal domain (Fig. 5D; Movie S2), the fragments highly deficient in hydrophobic residues (35% in KID, 38% in JMR and 24% in C-term), and composed of charged and polar residues. The high-amplitude motion of C-terminal describes its two extreme positions, either at the KID or at the C-lobe, and a large number of intermediate conformations. The PCA modes reveal significant KID dynamics with respect to the KD, and demonstrate the internal movements within the domain.

The PCA modes, which are calculated for each structural domain after the least-square fitting of the MD conformations to the *average conformation* of the respective domain as a reference, reveal the same details on intra-domain intrinsic motion (Fig. 5D,G; Movie S3). Focusing on KID, we observed that its structural fragments show a different degree of mobility – from little (αH1 and 3_10_-H4) to very extensive (the metastable helices αH2 and 3_10_-H3, and their adjacent linkers) (Fig. 5G; Movie S4).

The cross-correlation map of the dynamics that is computed for the all Cα-atom pairs shows a fractal-like pattern within each structural domain characterising a highly coupled motion in N- and in C-lobes (positive correlation) of KD (Fig. 5C). The cross-correlation pattern of the C-lobe shows six separated blocks exhibiting highly connected (positively correlated) intra-domain motions, and demonstrates dynamic coupling with the KID fragments and with the αE-helix bordering the N-lobe and the KID. The A-loop acts as a cleft that partially separate two highly correlated sub-regions of C-lobe and as an item contributing to the highly coupled motion with the N- and C-lobes.

### Conformational variability of KID

The cross-correlations computed for the KID Cα-atom pairs resulted in a well-ordered, chess-board like pattern showing that the dynamics of all KID structural fragments, the αH- and 3_10_-helices, and the coiled linkers, are tightly coupled (Fig. 5F). As these highly correlated motions involve the phosphotyrosine residues positioned on α-helices (Y703 and Y721), on 3_10_-helix (Y730) and on the loop linking two (Y747) (Fig. 5E; Movie S4), we focused on the conformational variations of these residues.

**Figure 6:**
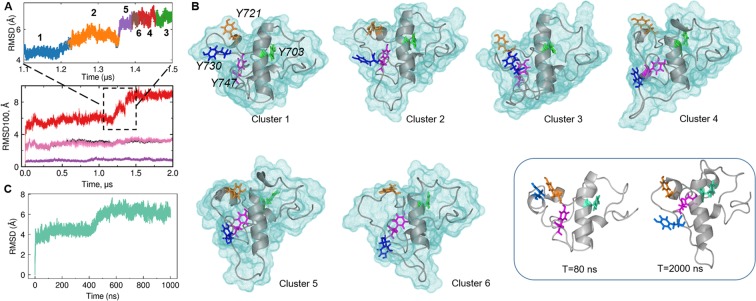
Conformational variability of KID. (**A**) Top: The KID conformations (in the range of 1.1–1.5 µs) were grouped (by k-means) into 6 RMSD-based clusters (Methods) distinguished by colour and numbered according to the cluster population (in descending order) with the time of simulation (in brackets) where the *representative conformations* were picked: **1** (1.15 µs)> **2** (1.30 µs)> **5** (1.37 µs); **6** (1.40 µs)> **4** (1.43 µs)> **3** (1.48 µs). Bottom: RMSD100 computed for all Cα-atoms (black) and for each domain individually: KD, KID and two-domain (KD and KID) (violet, red and pink respectively) after least-squares fitting of MD conformations on a region of interest (the *initial conformation* at t = 0 ns as a reference) and rescaled independently of the size of analyzed domain (see Methods). (**B**) *Representative conformations* of each cluster (KIT surface is shown) and two conformations, picked at t = 80 and 2000 ns, are shown in grey with the tyrosine residues as sticks. The numbering and colour of each tyrosine was maintained in all conformations. (**C**) RMSDs calculated for the Cα-atoms from the MD conformations of the isolated KID with respect to its initial conformation.

Since the main atomic variations in KID were captured in the MD conformations generated from 1150 to 1450 ns (the RMSDs transition range), we explored this data to obtain information on the intrinsic dynamics of KID during the transition. First, the KID conformations from the slope range were clustered (k-means) into six different sub-sets and the *representative conformations* from these clusters were analyzed (Fig. 6A). We observed that KID includes the quasi-rigid part composed of the αH1- and 3_10_H4-helices and the loops linking these helices to N- and C-lobes of KD respectively, and the highly variable region formed by the metastable αH2- and 3_10_H3-helices with their linkers (Fig. 6B).

To probe a *genuine* transition, we performed a 1-µs MD simulation of an isolated KID (polypeptide F689–D768) under the same physical conditions which were used for the full-length cytoplasmic domain. The RMSD profile of the isolated KID is similar to those observed for the KID integrated into the kinase domain of KIT (Fig. 6C). Specifically, the RMSD curve exhibits a slope from 420 to 550 ns, preceded and followed by the two stable regions with the RMSD mean values of 4.2 and 6 Å respectively.

This examination has proven that the KID transition is a reproducible event that is independent of its context, either as a remote polypeptide or as the KIT sub-domain. Detailed analysis of the conformational features of KID in different contexts (the native and phosphorylated states of a remote polypeptide in linear and cyclic forms) will be published as soon as possible.

## Discussion

The structural models of KIT, which are obtained by integration of the *ab initio* KID models into the kinase domain X-ray structure and then are studied by MD simulation, have led to assignment of the most likely the full-length KIT cytoplasmic domain model in the inactive state. The model choice is based on statistically valid metrics that characterise the protein’s biophysical and structural properties. Indeed, in the model **M3**, the experimentally determined unit composed of N- and C-lobes, and the generated *de novo* KID show the lowest RMSD and RMSF values with minimal variations. Moreover, the secondary structure assignment indicates that replacement of the ‘pseudo-KID’ by the ‘functional KID’ in this model does not influence the kinase domain structure. The distance between residues F689 and D768 is highly conserved over the MD simulation for two models (**M3** and **M4)**, however, only in **M3**, does its value correspond to that observed in all KIT structures. Finally, the KID’s radius of gyration in **M3** reveals its more tightly packed conformation relative to other models. We therefore concluded that **M3** is the most structurally realistic of the four candidate models, and this model was chosen for further study of the molecular dynamics of the full-length KIT cytoplasmic region in the inactive autoinhibited state.

The 2-µs MD simulation of this model has provided insight into the conformational features of KIT. This is a dynamics machine that undergoes atomic fluctuations, including side chain rotations and collective domain movements, that are required to perform its biological functions – regulation of catalytic activity and control of the signalling cascade. We have shown that the high conformational variability of KIT is furnished mainly by the JMR, C-terminal and KID, which are the principle platforms of post-translational processes. The intrinsic motion in KIT showed a high level of intra- and inter-domain coupling. Whereas the correlated motion in the N-lobe demonstrates a high level of regulation mechanisms involving the structural fragments implicated in kinase activity (P-loop, C-helix, C- and A-loops), which are observed here and have been previously described^21–23^, the functional context of motion of the other KIT domains, such as the C-lobe and KID, is obscure. There is obviously potential value in delineating larger dynamic systems that can provide a higher-level description of functional motions, such as large-scale conformational changes upon post-translational modification. The delivered dynamic model of KIT may be used in such modelling, e.g. the KIT phosphorylation effects and the KIT-partner(s) interactions.

Focusing on KID dynamics, we have observed a significant conformational plasticity of KID, which is especially evidenced during the transition. This KID transition is a complex multi-step process that is presented as a number of detectable intermediate conformations. KID displays a large intrinsic motion leading to a significant structural/conformational reorganization revealed as (i) folding/unfolding of the αH2- and 3_10_H3-helices, (ii) alternative positioning of these helices with respect to the most stable αH1-helix and (iii) highly varied conformation of the extended coiled linkers. Two sets of KID conformations, before the slope and after the slope, are almost stable (RMSD of 1–1.5 Å) and demonstrate the highly different KID conformations (Fig. 6B). These conformational features classify KID as a protein hybrid that contains both intrinsically disordered and ordered regions^24^.

Knowledge of the structural and conformational variability of KID that contains multiple phosphorylation sites is the mandatory requirement for a study of the reversible arrangement of the specific binding site(s) of the signalling proteins. It is interesting that in all KID conformations, the tyrosine residues, that are described in the literature as the functional phosphorylation sites (Y703, a phosphotyrosine of the quasi-rigid αH1-helix, and Y721 and Y730 positioned on the metastable αH2- and 3_10_H3-helices respectively), demonstrate highly variable orientations, in which these residues are always *solvent*-*accessible*, and consequently, easily available for post-transduction events. On the contrary, Y747, which is a tyrosine with an unknown function, is located inside the KID in a buried position close to the αH1-helix. Inspection of its contacts with the neighboring atoms showed that such a position of Y747 is stabilized by multiple non-covalent interactions (H-bonds, π-effects, stacking and hydrophobic interactions) with the αH1-helix residues. Moreover, each Y747 conformation is maintained by transiently formed inter-residue interactions (Figs. 6; S5). The authors suggest that Y747 plays a significant structural role in stabilization of the different meta-stable KID conformations through its strong transient non-covalent interactions. Such structural features of the KID tyrosine residues demonstrate the consistency of the established model with respect to known biological properties of KID and therefore tends to validate the physical correctness of the model.

## Methods

### Experimental model and subject details

The crystallographic structure retrieved from the PDB (PDB ID: 1T45) (resolution of 1.9 Å)^20^ of the CD of KIT (inactive conformation) contains the sequence Y547-V936 as lacking KID (Q694-T753 aas) and 41 C-terminal residues. The full sequence of human KIT (Blast sequence *P10721*) was used to predict the secondary structure of KID and C-terminal, and for estimation of the disordered regions.

## Method Details

### Molecular modelling

#### Secondary structure prediction

To predict the secondary structure of KID and the C-terminal fragment, four conceptually independent methods were used − GOR4^25^, PROFsec^26^, PsiPred^27^ and Jpred4^28^.

#### Prediction of disordered regions

To predict the disordered regions of the KID sequence we used the Multilayered fusion-based disorder predictor (MFDp2)^29^ that combines three complementary disorder predictors (IUpred, Disopred and DISOclust).

#### *De novo* modelling of KIT 3D structure

Modelling of the full-length KIT CD was realized as a multi-steps procedure by combining the structural data (1T45) and *de novo* modelling of KID (*ex situ*) and C-terminal. For KID modelling, we used the KID sequence elongated at each extremity by adding the 10 adjacent amino acids from the N- and the C-lobes of the kinase domain, F689–D768. First, two thousand *ex situ* pro-models were generated with ROSETTA that utilizes sequence-similar fragments by searching against three-dimensional structure databases followed by a fragment assembly using empirical intermolecular force fields^30^. The generated pro-models were further filtered using a distance *d* between residues F689 and D768 of 9.9 ± 1.0 Å (as observed in structure 1T45), resulting in a set of 70 models. The pre-selected suitable pro-models of KID were inserted into the X-ray structure 1T45, producing 70 pro-models, which were completed with the 41 residues of the C-terminal domain (K936-V976 aas) built *ab initio* with MODELLER^31^. All models showing structural aberrations (*i.e*., intramolecular “nodes”) were identified by visual inspection with PyMol and eliminated, and the 26 models having the highest DOPE scores (*i.e*., lowest energy)^32^ were considered as plausible and were clustered^33^ according to their secondary structure similarity (DSSP^34^). Finally, a hierarchical clustering, which was applied to the score matrix to calculate the distance *d* between the newly formed cluster *u* and each cluster *v* was obtained using a farthest point algorithm^35^ (https://scipy.org/):

1 $$d(u,v)=max(dist(u[i],v[j]))$$

for each element *i* in cluster *u* and *j* in cluster *v*.

The four most distinct models, **M1–M4**, were selected for MD simulations. The stereochemical quality of these models was assessed by Procheck^36^, revealing more than 95% of the non-glycine/non-proline residues in all models to have the dihedral angles in the most favored and the permitted regions of the Ramachandran plot, as is expected for good models.

#### Molecular dynamics simulation

System preparation. For MD simulation, models **M1**–**M4** were prepared with the LEAP module^37^ of AMBER 15 using *ff99SB* all-atom force filed parameter set: (i) hydrogen atoms were added, (ii) covalent bond orders were assigned, (iii) protonation states of amino acids were assigned based on their solution for pK values at neutral pH, histidine residues were considered neutral and protonated on ε-nitrogens, (vi) Na^+^ counter-ion was added to neutralize the protein charge, (v) each protein is composed of 6,358 atoms (**M1–M4**) was solvated with explicit TIP3P water molecules in a periodic rectangular box with at least 12 *Å* distance between the proteins and the boundary of the water box. The total number of atoms in the systems (protein, water molecules and counter ion) varied from 62,930 to 70,130 for the **M1**–**M4** models.

Set up of the systems. The set-up of the systems was performed with the SANDER module^38^ of AMBER 14. First, each system was minimized successively using the steepest descent and conjugate gradient algorithms as follows: (i) 10,000 minimization steps where the water molecules have fixed protein atoms, (ii) 10,000 minimization steps where the protein backbone is fixed to allow protein side chains to relax, and (iii) 10,000 minimization steps without any constraint on the system. After relaxation, each system was gradually heated from 10 to 310 K at constant volume using the Berendsen thermostat^39^ while restraining the solute Cα atoms by 10 kcal/mol/Å^2^. Thereafter, the system was equilibrated for 100 ps at constant volume (NVT) and for a further 100 ps at constant pressure (NPT) maintained by a Langevin piston^40^. The velocities were reassigned according to the Maxwell-Boltzmann distribution. Finally, the restraints were removed and each system was equilibrated for a final 100-ps run.

Production of the trajectories. For each equilibrated system, **M1–M4**, two MD simulations of 100 ns (replica) were performed with the PMEMD module of AMBER 15^41^ (GPU-accelerated version) was run on a hybrid server (Ubuntu, LTS 14.04, 252 GB RAM, 2x CPU Intel Xeon E5-2680 (16 cores, 32 threads, 2,7–3,5 GHz) et Nvidia GTX 780ti). The temperature was kept at 310 K (Berendsen thermostat), and pressure at 1 bar (Langevin piston coupling algorithm). The SHAKE algorithm was used to freeze the covalent bonds involving hydrogen atoms, allowing for an integration time step of 2.0 fs. Long-range electrostatic interactions were treated by the Particle Mesh Ewald method^42^. Coordinates were recorded every 1 ps. Based on a similar behavior of RMSD values of both replicas for each model, the MD simulations were extended to 500 ns for all models, a single trajectory for **M1, M2** and **M4** and two trajectories for **M3**, one of them was further extended to 2 µs.

Analysis of the MD trajectories. Unless otherwise stated, all recorded MD trajectories were analyzed (RMSFs, RMSDs, DSSP, clustering) with the standard routines CPPTRAJ of AMBER 16 Suite^43^. The RSMD and RMSF values were calculated for the Cα atoms using the initial model (at t = 0 ns) as a reference. All analysis was performed on the MD trajectories considering either all simulation or the production part of the simulation, which was after removal of non-equilibrated conformations (0–80 ns) as was shown by the RMSDs, and after tleast-square fitting^44,45^ of the MD conformations for a region of interest, thus removing rigid-body motion from the analysis. Secondary structures were assigned every 10 ps using DSSP^34^ integrated into CPPTRAJ and evaluated for the total structural tendancy over all residues for each secondary structure type. Visual inspection of conformations and figure preparation was made with PyMOL^46^. The gyration radii were computed with the python package MDAnalysis (www.mdanalysis.org).

Principal components (PCs) analysis, an unsupervised popular dimension reduction technique, was applied to found patterns in high-dimensional data. The PCs describe concerted atomic displacements in protein and can highlight major conformational changes^47^. The PCs are obtained by a diagonilization of the data covariance matrix *C* (1).

2 $$C=V\varLambda {V}^{T}$$

The diagonal matrix $\varLambda$ contains the eigenvalues as diagonal entries and the matrix *V* contains the corresponding eigenvectors.

NThe normalized, size-independent RMSD (RMSD100) metric was used to compare RMSDs of different-sized domains^48^. The cross-correlation coefficient of the atomic fluctuations (Cα-atoms) that was obtained from the MD simulations was computed with the algorithm published in^49^. Clustering of the trajectory data was performed using the k-means algorithm with the Euclidean distance as a similarity measure. The optimal number of clusters was determined by running the clustering several times with a different number of clusters for each run and comparing the solutions using the Davies-Bouldin and the Calinski-Harabasz indices as quality measure metrics. RMSDs were calculated for each KIT domain individually after least-square fitting of the MD conformations on the initial KIT domain conformation.

The VMD 1.9.3 program^50^ was used to prepare the protein MD animations. To visualize the motions along the principal components, the Normal Mode Wizard (NMWiz) plugin^51^ that is distributed with VMD was utilized.

## Supplementary information

Supplementary Information.

Movie S1.

Movie S2.

Movie S3.

Movie S4.

## References

[1] Downward J (2001). The ins and outs of signalling. Nature.

[2] Lemmon MA, Schlessinger J (2010). Cell signaling by receptor tyrosine kinases. Cell.

[3] Du Z, Lovly CM (2018). Mechanisms of receptor tyrosine kinase activation in cancer. Mol. cancer.

[4] Bennasroune A, Gardin A, Aunis D, Cremel G, Hubert P (2004). Tyrosine kinase receptors as attractive targets of cancer therapy. Crit. Rev. oncology/hematology.

[5] Schlessinger, J. Receptor tyrosine kinases: legacy of the first two decades. *Cold Spring Harbor perspectives in biology* **6**, 10.1101/cshperspect.a008912 (2014). 10.1101/cshperspect.a008912 PMC3949355 24591517

[6] Berman HM (2000). The Protein Data Bank and the challenge of structural genomics. Nat. Struct. Biol..

[7] Locascio LE, Donoghue DJ (2013). KIDs rule: regulatory phosphorylation of RTKs. Trends Biochem. Sci..

[8] Opatowsky Y (2014). Structure, domain organization, and different conformational states of stem cell factor-induced intact KIT dimers. Proc. Natl Acad. Sci..

[9] Reshetnyak AV (2015). The strength and cooperativity of KIT ectodomain contacts determine normal ligand-dependent stimulation or oncogenic activation in cancer. Mol. Cell.

[10] Zhang HM (2010). Drug binding and resistance mechanism of KIT tyrosine kinase revealed by hydrogen/deuterium exchange FTICR mass spectrometry. Protein science: a Publ. Protein Soc..

[11] Mol CD (2003). Structure of a c-kit product complex reveals the basis for kinase transactivation. J. Biol. Chem..

[12] Gajiwala KS (2009). KIT kinase mutants show unique mechanisms of drug resistance to imatinib and sunitinib in gastrointestinal stromal tumor patients. Proc. Natl Acad. Sci. USA.

[13] Ronnstrand L (2004). Signal transduction via the stem cell factor receptor/c-Kit. Cell. Mol. life sciences: CMLS.

[14] Chatron N (2017). Identification of the functional states of human vitamin K epoxide reductase from molecular dynamics simulations. RSC Adv..

[15] Kato, K., Nakayoshi, T., Fukuyoshi, S., Kurimoto, E. & Oda, A. Validation of Molecular Dynamics Simulations for Prediction of Three-Dimensional Structures of Small Proteins. *Molecules (Basel, Switzerland)* **22**, 10.3390/molecules22101716 (2017). 10.3390/molecules22101716 PMC6151455 29023395

[16] Piana S, Klepeis JL, Shaw DE (2014). Assessing the accuracy of physical models used in protein-folding simulations: quantitative evidence from long molecular dynamics simulations. Curr. Opin. Struct. Biol..

[17] Srivastava, A., Nagai, T., Srivastava, A., Miyashita, O. & Tama, F. Role of Computational Methods in Going beyond X-ray Crystallography to Explore Protein Structure and Dynamics. *International journal of molecular sciences* **19**, 10.3390/ijms19113401 (2018). 10.3390/ijms19113401 PMC6274748 30380757

[18] van der Geer P, Hunter T (1990). Identification of tyrosine 706 in the kinase insert as the major colony-stimulating factor 1 (CSF-1)-stimulated autophosphorylation site in the CSF-1 receptor in a murine macrophage cell line. Mol. Cell Biol..

[19] Barth P, Senes A (2016). Toward high-resolution computational design of the structure and function of helical membrane proteins. Nat. Struct. Mol. Biol..

[20] Mol CD (2004). Structural basis for the autoinhibition and STI-571 inhibition of c-Kit tyrosine kinase. J. Biol. Chem..

[21] Chauvot de Beauchêne I (2014). Hotspot mutations in KIT receptor differentially modulate its allosterically coupled conformational dynamics: impact on activation and drug sensitivity. PLoS computational Biol..

[22] Chauvot de Beauchêne, I. & Tchertanov, L. How missense mutations in receptors tyrosine kinases impact constitutive activity and alternate drug sensitivity: insights from molecular dynamics simulations. *Receptors & Clinical Investigation* **3** (2016).

[23] Laine E, Chauvot de Beauchêne I, Perahia I, Auclair D, Tchertanov C (2011). L. Mutation D816V alters the internal structure and dynamics of c-KIT receptor cytoplasmic region: implications for dimerization and activation mechanisms. PLoS Comput. Biol..

[24] Uversky, V. N. Intrinsically Disordered Proteins and Their “Mysterious” (Meta)Physics. *Frontiers in Physics* **7**, 10.3389/fphy.2019.00010 (2019).

[25] Garnier J, Gibrat JF, Robson B (1996). GOR method for predicting protein secondary structure from amino acid sequence. Methods enzymology.

[26] Rost B, Sander C (1994). Combining evolutionary information and neural networks to predict protein secondary structure. Proteins.

[27] Jones DT (1999). Protein secondary structure prediction based on position-specific scoring matrices. J. Mol. Biol..

[28] Drozdetskiy A, Cole C, Procter J, Barton GJ (2015). JPred4: a protein secondary structure prediction server. Nucleic Acids Res..

[29] Mizianty MJ, Peng Z, Kurgan L (2013). MFDp2: Accurate predictor of disorder in proteins by fusion of disorder probabilities, content and profiles. Intrinsically disordered proteins.

[30] Alford RF (2017). The Rosetta All-Atom Energy Function for Macromolecular Modeling and Design. J. Chem. theory computation.

[31] Webb B, Sali A (2017). Protein Structure Modeling with MODELLER. Methods Mol. Biol..

[32] Shen MY, Sali A (2006). Statistical potential for assessment and prediction of protein structures. Protein science: a Publ. Protein Soc..

[33] Volkert, L. G. & Stoffer, D. A. A comparison of sequence alignement algorithms for measuring secondary structure similarity, In *Proceeding of the 2004 IEEE Symposium on Computational Intelligence in Bioinformatics and Computational Biology (CIBCB’04), La Jolla*, California, October 7–8, pp. 182–189 (2004).

[34] Kabsch W, Sander C (1983). Dictionary of protein secondary structure: pattern recognition of hydrogen-bonded and geometrical features. Biopolymers.

[35] Voorhees H, Poggio T (1988). Computing texture boundaries from images. Nature.

[36] Laskowski RA, Rullmannn JA, MacArthur MW, Kaptein R, Thornton JM (1996). AQUA and PROCHECK-NMR: programs for checking the quality of protein structures solved by NMR. J. biomolecular NMR.

[37] Wang J, Wang W, Kollman PA, Case DA (2006). Automatic atom type and bond type perception in molecular mechanical calculations. J. Mol. Graph. Model..

[38] Kaus, J. W., Pierce, L. T., Walker, R. C. & McCammont, J. A. Improving the Efficiency of Free Energy Calculations in the Amber Molecular Dynamics Package. *Journal of chemical theory and computation* **9**, 10.1021/ct400340s (2013). 10.1021/ct400340s PMC3811123 24185531

[39] Peters EA, Goga N, Berendsen HJ (2014). Stochastic Dynamics with Correct Sampling for Constrained Systems. J. Chem. theory computation.

[40] Loncharich RJ, Brooks BR, Pastor RW (1992). Langevin dynamics of peptides: the frictional dependence of isomerization rates of N-acetylalanyl-N’-methylamide. Biopolymers.

[41] Case DA (2005). The Amber biomolecular simulation programs. J. computational Chem..

[42] Sagui C, Pedersen LG, Darden TA (2004). Towards an accurate representation of electrostatics in classical force fields: efficient implementation of multipolar interactions in biomolecular simulations. J. Chem. Phys..

[43] Roe DR, Cheatham TE (2013). PTRAJ and CPPTRAJ: Software for Processing and Analysis of Molecular Dynamics Trajectory Data. J. Chem. theory computation.

[44] Kabsch W (1976). A solution for the best rotation to relate two sets of vectors. Acta Crystallogr. Sect. A.

[45] Kabsch W (1978). A discussion of the solution for the best rotation to relate two sets of vectors. Acta Crystallogr. Sect. A.

[46] DeLano WL (2005). The case for open-source software in drug discovery. Drug. discovery today.

[47] Amadei A, Linssen AB, Berendsen HJ (1993). Essential dynamics of proteins. Proteins.

[48] Carugo O, Pongor S (2001). A normalized root-mean-square distance for comparing protein three-dimensional structures. Protein science: a Publ. Protein Soc..

[49] Hunenberger PH, Mark AE, van Gunsteren WF (1995). Fluctuation and cross-correlation analysis of protein motions observed in nanosecond molecular dynamics simulations. J. Mol. Biol..

[50] Humphrey W, Dalke A, Schulten K (1996). VMD: visual molecular dynamics. J. Mol. Graph..

[51] Bakan A (2014). Evol and ProDy for bridging protein sequence evolution and structural dynamics. Bioinformatics.

[52] Yuzawa S (2007). Structural Basis for Activation of the Receptor Tyrosine Kinase KIT by Stem Cell Factor. Cell.

