# Supplementary material for: The First 3D Model of the Full-Length KIT Cytoplasmic Domain Reveals a New Look for an Old Receptor
Source: Sci Rep. 2020 Mar 25;10:5401. doi: 10.1038/s41598-020-62460-7 (PMC7096506; doi:10.1038/s41598-020-62460-7)
Supplement: Supplementary file 1 — Supplementary Information. [file 41598_2020_62460_MOESM1_ESM.pdf]

The First 3D Model of the Full-Length KIT Cytoplasmic Domain Reveals a New Look for an Old Receptor

François Inizan, Myriam Hanna, Maxim Stolyarchuk,

Isaure Chauvot de Beauchêne and Luba Tchertanov

Supplementary Information:

Figures S1- S5 with the legends

Movies S1-S4: The legends

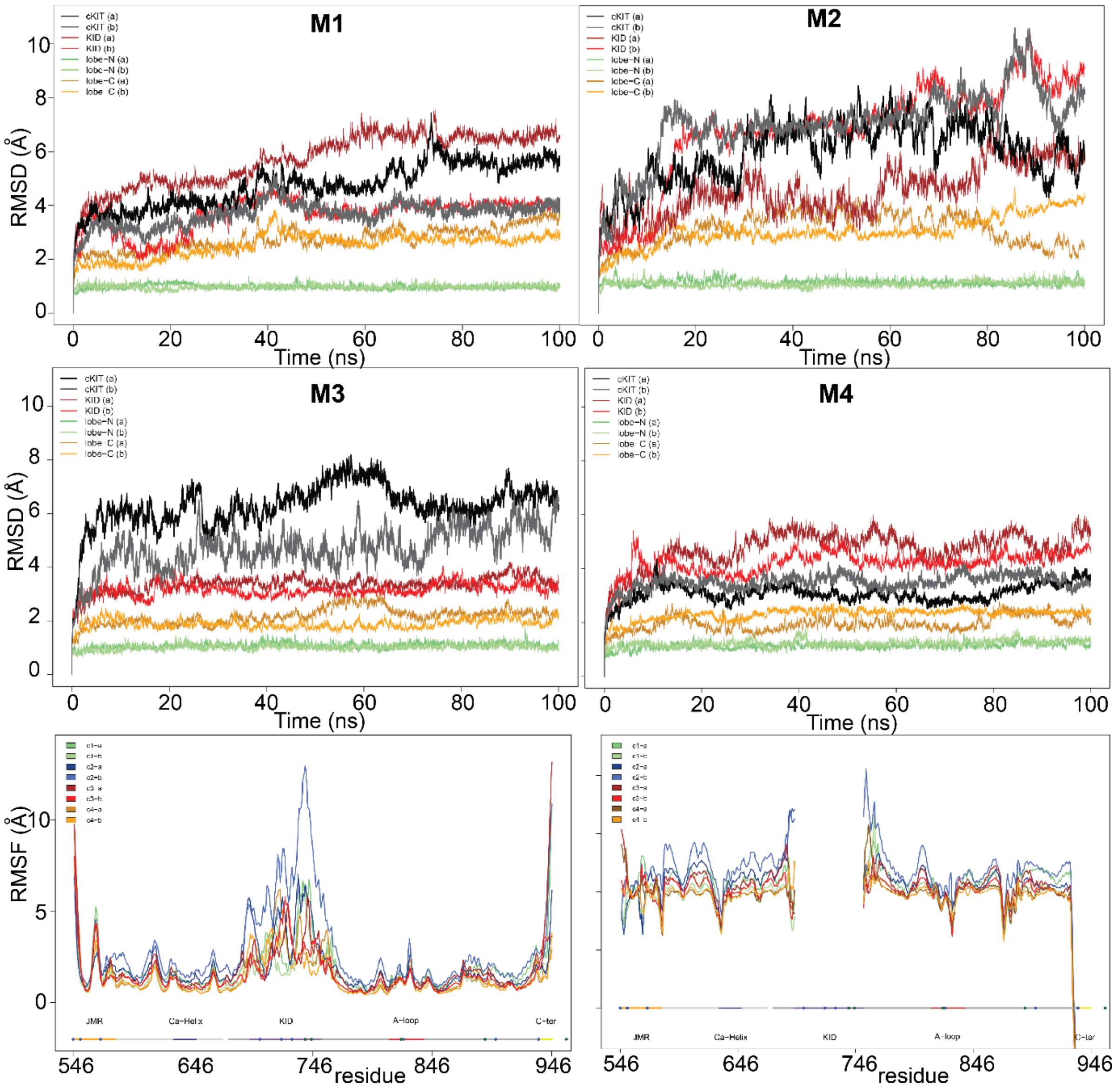

Figure S1. **MD simulations of candidate models (M1-M4) of the full-length KIT cytoplasmic domain**. (Top and middle panels): The root mean square deviations (RMSDs) from the initial coordinates computed for all Cα-atoms (black/grey), then only Cα- atoms from N-lobe (green/light green), C-lobe (orange/yellow) and KID (red/brow) for the **M1-M4** models during two 100 ns MD replicas, denoted as (**a**) and (**b**). (Bottom panel, in left): The root mean square fluctuations (RMSFs) computed on the Cα-atoms of **M1-M4** models for each replica, denoted as (**a**) and (**b**). (Bottom panel, in right): Difference of the RMSFs computed on the Cα-atoms of **M1 - M4** model for each replica, denoted as (**a**) and (**b**) relative to the MD from the crystallographic structure (template structure, PDB:1T45). Models **M1-M4** are denoted as ci, i=1, 2, 3, 4) in color code panels.

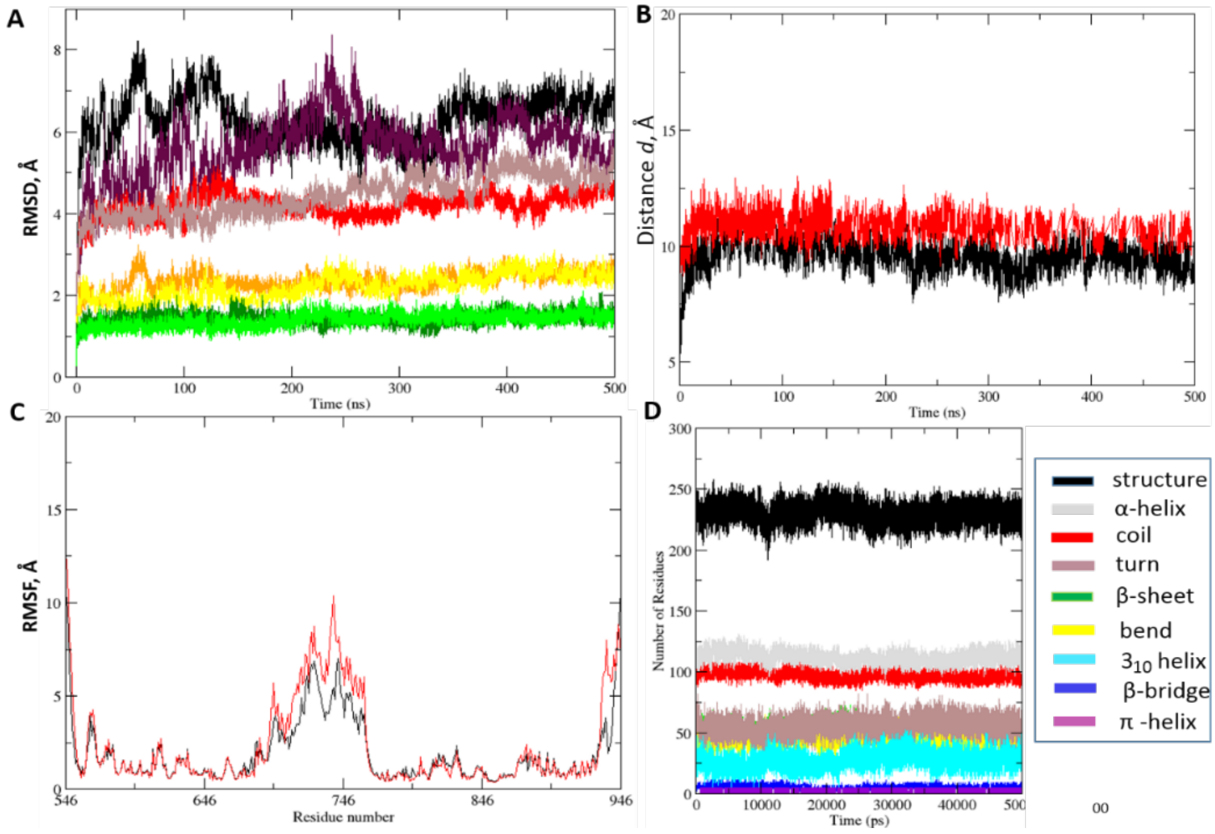

Figure S2. MD simulations of candidate model M3 of the full-length KIT cytoplasmic domain. (**A**) The root mean square deviations (RMSDs) from the initial coordinates computed for all Cα-atoms (black/brun), then only Cα- atoms from N-lobe (green/light green), C-lobe (orange/yellow) and KID (red/salmon) for the **M3** model during two 500-ns MD replicas. (**B**) Distance (*d*) between residues F689 and D768 over two MD trajectories of models **M3** (black/red). (**C**) RMSFs computed on the Cα-atoms of **M3** model for each replica (black/red). (**D**) Secondary structure in KIT **M3** model during the second MD replica. The position of the curves reflects the amount of secondary structure elements in the models, from a tiny occurrence [π-helix (violet) and β-bridge (blue)] to a prevalent [α-helix (grey), coil (red) and turn (brown)], and sum of folded structures in black.

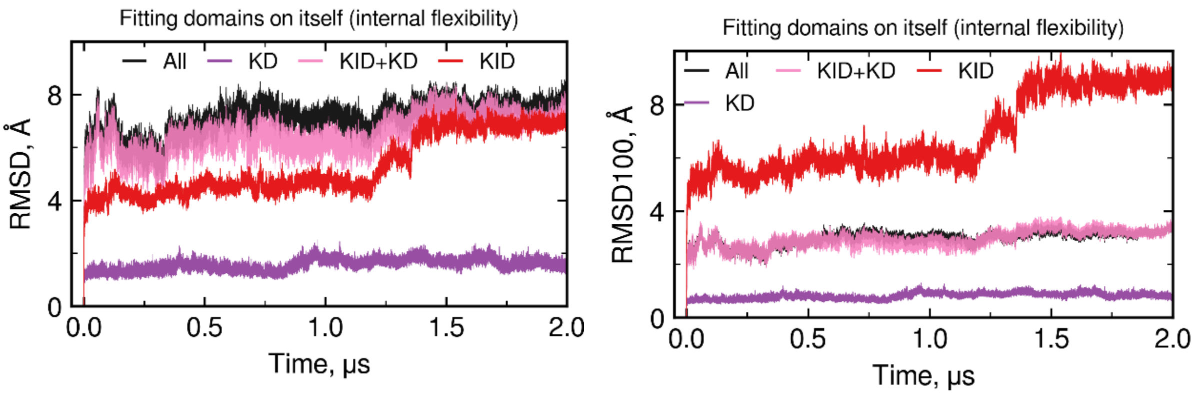

Figure S3. **Analysis of the MD conformations of KIT M3 model**. (Left) RMSDs calculated for all Cα-atoms (black), then only for Cα atoms from the partial protein composed of KD and KID (rose); the KD (violet) and the KID (red) after the least-square fitting of MD conformations on a region of interest (the *initial conformation* at t= 0 ns as a reference). (Right) RMSDs-100 were computed for all Cα-atoms (black), then only for Cα atoms from the partial protein composed of KD and KID (rose); the KD (violet) and the KID (red) after the least-square fitting of MD conformations on a region of interest (the *initial conformation* at t= 0 ns as a reference) and rescaled independently of a size of analyzed domain (Carugo & Pongor, 2001).

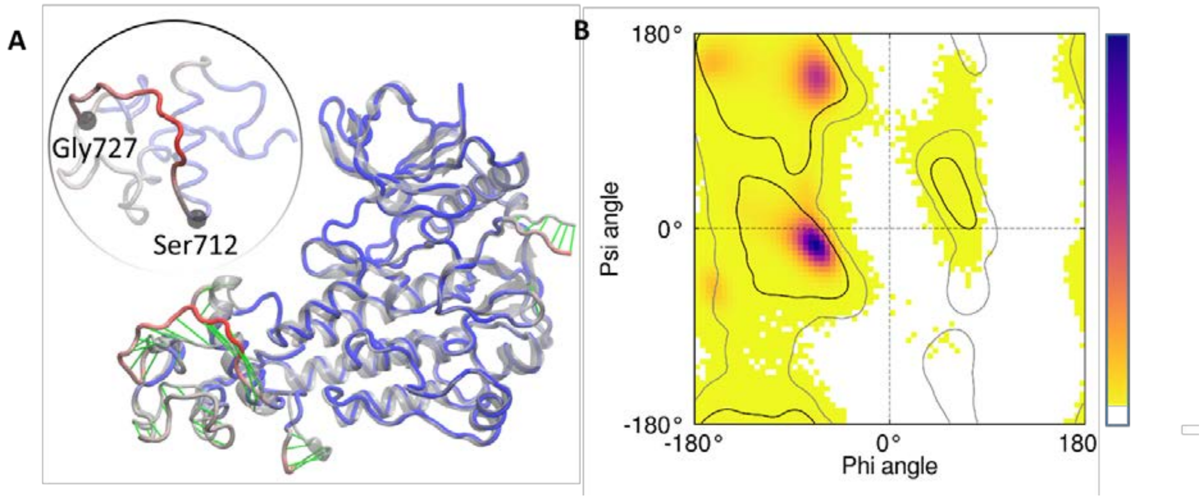

Figure S4. **Evaluation reliability for 3-dimensional KID conformations**. (**A**) The most variable fragments (in red) of KIT were defined from the 1st PCA mode. The green lines show the displacement between the initial (in grey) and final (in blue) conformations. (**B**) The Ramachandran map (Φ and ψ distribution) of non-glycine, non proline-residues of the backbone of the most flexible fragment of KID (Ser712 − Gly727) within the range from 1190 to 1365 ns.

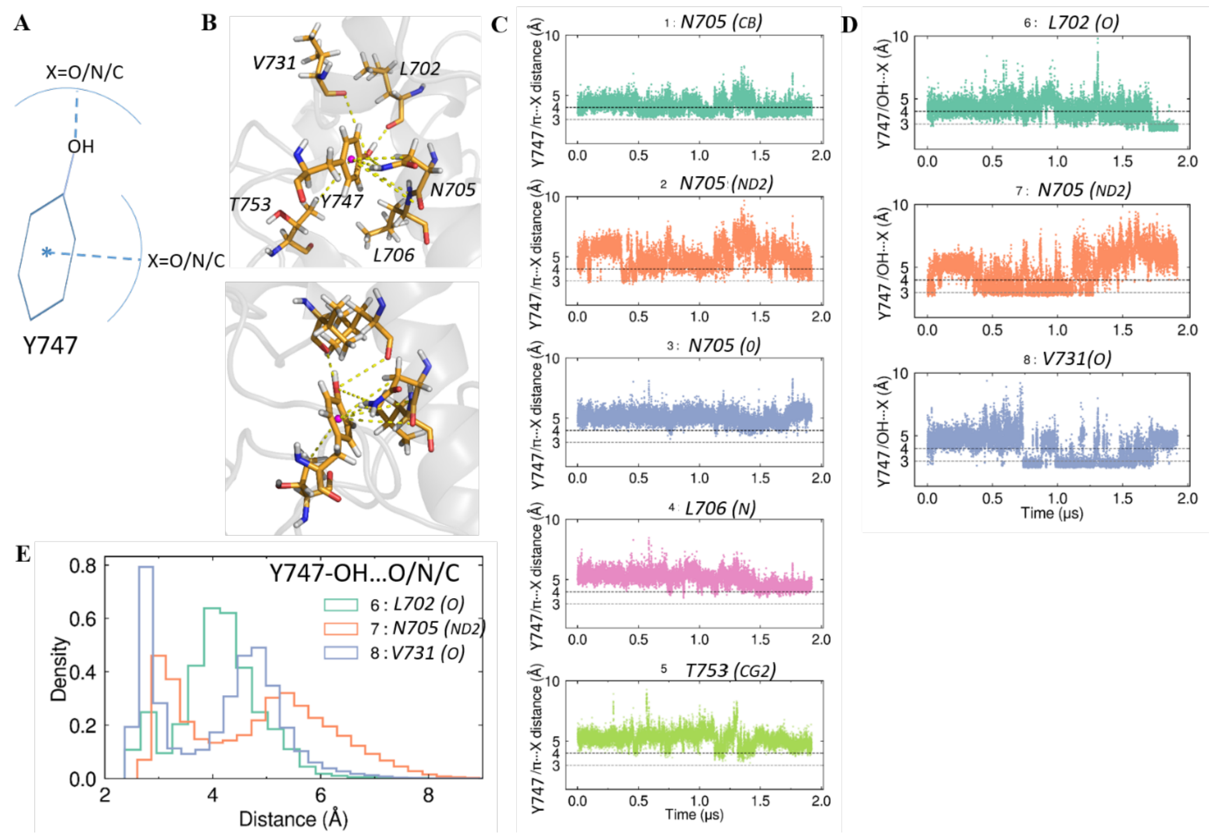

Figure S5. **The non-covalent contacts of Y747 with neighbor residues**. (**A**) Contacts were computed from the centroid determined on the aromatic ring of tyrosine shown as asterisk (*), and from the oxygen atom of OH-group (as the origin) to any atom, X = O/N/C (as the target) in the radii of 10 Å. (**B**) The inter-residues contacts formed by Y747 with the neighboring residues. Protein is shown as cartoon with the interacting residues in sticks (two projections are shown). (**C**) The distances between Y747 and its contact residues observed during the MD simulation.

Movie S1. MD trajectory from 1150 to 1450 ns (the RMSD slope range) demonstrates the transition between different KID configurations.

Movie S2. The PCA 1st mode of KIT calculated for all Cα-atoms after least-square fitting of MD conformations to the *average conformation* of KIT as a reference.

Movie S3. The PCA 1st mode of the kinase domain (KD) calculated for Cα-atoms after least-square fitting of MD conformations to the *average conformation* of KD as a reference.

Movie S4. The PCA 1st mode of the kinase insert domain (KID) calculated for Cα-atoms after least-square fitting of MD conformations to the *average conformation* of KID as a reference.
